# The effect of drinking milk containing conjugated linoleic acid on fecal microbiological profile, enzymatic activity, and fecal characteristics in humans

**DOI:** 10.1186/1475-2891-6-15

**Published:** 2007-07-09

**Authors:** Edward R Farnworth, Yvan P Chouinard, Helene Jacques, Sudha Venkatramanan, Akier A Maf, Sabrina Defnoun, Peter JH Jones

**Affiliations:** 1 Food Research and Development Centre, Agriculture Canada, Saint Hyacinthe, Quebec, Canada; 2 Departments of Animal Science and Food Science and Nutrition, Laval University, Quebec City, Quebec, Canada; 3 School of Dietetics and Human Nutrition, McGill University, Montreal, Quebec, Canada

## Abstract

**Background:**

The primary objective was to determine whether consumption of conjugated linoleic acids (CLAs) affected the fecal microbiota composition, fecal enzyme activity or fecal composition.

**Methods:**

Human subjects consumed (1 *L*/day) cows' milk (4% fat) containing (5 mg/g fat) *cis*-9, *trans*-11 CLA (CONT), (32 mg/g fat) cis-9, *trans*-11 CLA (NAT) and (32 mg/g fat) *trans*-10, *cis*-12 CLA and *cis*-9, *trans*-11 CLA (SYN) for 8 weeks, in addition to their normal diet. Milk feeding periods were separated by 4 week washout periods. Fecal samples were obtained at the beginning (day 0) and the end (day 56) of each milk feeding period. Fecal samples were analysed for microbiological profile, enzyme activity, pH and short chain fatty acid content.

**Results:**

Samples taken at day 0 and day 56 indicated that the numbers of *lactobacilli *and *bifidobacteria *significantly decreased after consumption of all experimental milks; total aerobes, total anaerobes, enterobacteria, and enterococci + streptococci did not change. At day 56, the activities of β-glucosidase, nitroreductase, and urease enzymes had decreased compared to samples taken on day 0 for all treatments. β-glucuronidase activity did not change. Fecal pH and ammonia content did not change.

**Conclusion:**

It was concluded that observed changes could have been attributed to increased milk intake; no differences could be attributed to consumption of the different CLAs.

## Background

A variety of positional and geometrical isomers of linoleic acid are included in the general term conjugated linoleic acids (CLAs). Rumen bacteria produce CLAs [1,2] from dietary linoleic acid, and as a result, red meats and dairy products are the main sources of *cis*-9, *trans*-11 CLA in the human diet. In foods, CLAs are in the triglyceride form.

Experiments in which humans were fed CLAs have reported loss of body weight [3], reductions of % body fat and sagittal abdominal diameter [3-5], and a positive impact on some coronary artery disease risk factors [6]. Various mechanisms have been presented to explain the mode of action of CLAs, either in animals or humans [4-9].

The gastrointestinal tract is inhabited by a large and diverse microbiota [10,11]. This microbial population is relatively stable, but changes occur due to age, disease status, use of medications such as antibiotics, and diet [12-14]. It has been speculated that changes to the intestinal microbiota could explain alterations in lipid metabolism [15,16]. *In vitro *experiments have shown that CLAs can inhibit growth of some bacteria and alter bacteria membrane lipid composition [17]. However, very few studies have been published in which the effects of the lipid component of the diet on the gastrointestinal (GI) tract microbiota have been reported [18-20].

It is believed that the microbiota that inhabits the GI tract influences a wide variety of digestive, metabolic, and immune functions [21]. Carman *et al*. [22] pointed out that changes in the intestinal microflora population may not be easy to achieve, but in any case, it is changes in 'microflora associated characteristics' (MACs) that are better indicators of effects of diet on the host. A change in enzymatic activity may be one MAC that has long term implications on health. The activities of various fecal enzymes have been reported to be influenced by dietary fat [23], carbohydrates [23-27], and consumed bacteria [25,28,29].

β-glucuronidase activity in feces comes from *Bacteriodes *and other bacteria. β-glucuronidase hydrolyzes a variety of glucuronides, liberating carcinogenic aglycones. Fecal β-glucosidase activity also comes mainly from *Bacteriodes*, but many streptococci and lactobacilli also have high β-glucosidase activity. β-glucosidase is responsible for the hydrolysis of plant β-glycosides, releasing into the intestinal lumen aglycones which are mutagenic and carcinogenic [30-32]. Nitroreductase enzyme acts on aromatic nitro-compounds resulting in the formation of harmful amines [33]. Urease enzyme can act on urea releasing ammonia and carbon dioxide; high urease activities have been found in some *Eubacteria *and *Peptococcus *bacteria [34]. Ammonia has been shown to promote the growth of tumors in the colon; it facilitates the growth of pathogenic bacteria and contributes to mucosal tissue damage [35]. Decreases in fecal β-glucuronidase, β-glucosidase, nitroreductase and urease activities are thus considered desirable because of their links to the production of carcinogens [36].

Goldin & Gorbach [37] reported that fecal β-glucuronidase, nitroreductase and azoreductase enzyme activities did not change when human subjects consumed 500 ml of low fat milk per day for 30 days. Conversely, in one Yakult feeding trial reported by Tanaka [38], β-glucuronidase enzyme activity decreased in 4 of 10 control subjects, β-glucosidase enzyme activity decreased in 3 of 10 control subjects, and reductase enzyme activity decreased in 3 of 10 control subjects consuming unfermented milk (240 ml/day) compared to pre-experiment values. In a second trail, the reductions of β-glucuronidase and β-glucosidase were statistically significant in subjects consuming unfermented milk (300 ml/day). Feeding lactose (20 g/day or 40/day) did not affect fecal β-glucosidase or β-glucuronidase activities in elderly subjects [24].

Lactobacilli along with bifidobacteria have received much attention as ingredients in probiotic products [39-41]. Feeding milk containing lactobacilli has been shown to successfully increase lactobacilli numbers [42,43]. However, feeding just lactose 20 g/day or 40 g/day has been shown to reduce significantly lactobacilli numbers [24].

It is apparent that CLAs are potent bioactive lipid ingredients in many foods. As CLAs pass down through the GI tract, they may be bringing about changes to the intestinal microbiota, which may in turn be contributing to their whole body effects.

This study was undertaken to determine whether the consumption of CLAs effected the intestinal microbiota population composition and function. Fecal samples were collected from subjects who had consumed different forms and amounts of CLA to determine whether the consumption of the experimental milks affected the numbers of various fecal bacteria, fecal enzyme activity or fecal composition. Data presented here were obtained during a larger experiment in which the effects of CLA consumption on lipid metabolism and body composition were studied.

## Methods

### Subject selection

Selection criteria for subjects in the study were: moderately hyperlipidemic (LDL cholesterol > 2.5 mmol/L), overweight (BMI 25–30 kg/m^2^) men and women between the ages of 30 and 60. Volunteers with thyroid disease, diabetes mellitus, kidney disease, or liver disease, who smoked, who had previous symptoms of lactose intolerance, consumed large amounts of alcohol or who were taking antibiotics were excluded.

Twice during the experiment, subjects filled out a questionnaire to determine their normal consumption of milk and dairy products.

### Milk samples

Three types of milk were used in the feeding trial: 4% fat homogenized cows' milk, containing 5 mg/g fat *cis*-9, *trans*-11 CLA (CONT); 4% fat homogenized cows' milk naturally enriched in *cis*-9, *trans*-11 CLA (32 mg/g fat) by feeding cows with sunflower oil (NAT) or 4% fat homogenized cows' milk that was enriched with *trans*-10, *cis*-12 CLA (32 mg/g fat) and *cis*-9, *trans*-11 CLA (32 mg/g fat) in the form of triglycerides (Natural Co., Norway), (SYN).

For the analysis of milk fatty acids, methyl esters were prepared by base-catalyzed transmethylation according to the method of Chouinard et al.[44]. Fatty acid analyses were carried out with a gas chromatograph (HP 5890A Series II, Hewlett Packard, Palo Alto, CA) equipped with a 100-m CP-Sil 88 capillary column (i.d., 0.25 mm; film thickness, 0.20 μm; Chrompack, Middelburg, the Netherlands) and a flame ionization detector. At the time of the sample injection the column temperature was 80°C for 1 min, then ramped at 2°C/min to 215°C and maintained for 30 min. Inlet and detector temperatures were 220°C and 230°C, respectively. The split ratio was 100:1. The flow rate for hydrogen carrier gas was 1 mL/min. Fatty acid peaks were identified, quantified and the gas chromatograph calibrated using pure methyl ester standards (Nu Chek Prep, Elysian, MN).

### Experimental design

The experiment was a three feeding phase (8 weeks each), cross-over design separated by 2 washout periods (4 weeks each). The single-blind clinical trial was carried out at the Mary Emily Clinical Research Unit, McGill University. In each feeding phase, one third of the subjects consumed either 1 L/day of CONT, NAT or SYN. The type of experimental milk consumed was changed in each feeding phase, until all subjects had consumed all three milks. Milk samples were coded; subjects did not know the code. Fecal samples were collected on days 0 and 56 of each feeding phase. All samples were identified by subject number only until analyses were complete.

### Microbiological analyses

Fecal samples were immediately refrigerated after collection and then they were mixed with a storage solution (pH 7.2) consisting of (per L): 1 g yeast extract, 1 g KH_2_PO_4_, 0.15 g K_2_HPO_4_, 0.15 g NHCl, 1 g NaCl, 0.6 g MgCl_2_6H_2_O, 0.1 g KCl, and 0.5 g cysteine-HCl, and stored at -20°C until enumeration.

Aliquots of diluted fecal samples were spread on the following aqueous agar media: Columbia blood agar (CBA) media (Oxoid Company, Basingstoke, UK) containing 5% sterile defibrinated blood (Quélab, Montreal, QC, Canada) incubated for total anaerobes; Columbia agar base (BBL, Becton Dickinson, Cockeysville, MD, USA) 950 mL containing lactose 5 g/L and cysteine hydrochloride 0.5 g/L and 50 mL NPNL stock containing neomycin (Sigma, St. Louis, MO, USA) 100 mg/L, paromomycin sulphate (Sigma) 200 mg/L for bifidobacteria; 500 mL Difco Lactobacilli MRS broth (Becton Dickenson Co., Sparkes MD, USA) containing cysteine hydrochloride 0.5 g/L, bromocresol green (Sigma) 0.05 g/L – pH 5.0, 10 mL vancomycin hydrochloride (Sigma) solution, 500 mL agar (Difco, Detroit, MI) 40 g/L for lactobacilli; MacConkey agar (Difco) for enterobacteriaceae; m-Enterococcus agar (Difco) for enterococci and streptococci; sulphite polymyxin milk (SPM) agar 930 mL containing tryptone (Difco) 15 g/L, yeast extract (Difco) 10 g/L, ferric citrate (Sigma) 0.5 g/L, agar (Difco) 18 g/L, 5 mL 5% Na_2_SO_3_, 10 mL 0.1% colistin sulphate (Sigma), 4 mL 1% neutral red solution (supplier), 50 mL sterile whole cows' milk for clostridia; Schaedler agar (Becton Dickinson, Sparks, MD) for total aerobes. Microbial data were expressed as log CFU/g wet feces.

### Enzyme assays

Thawed fecal samples were homogenized (Basic T25; IKA Co., Wilmington, NC, USA) in phosphate buffer (0.1 M; pH 7.0), sonicated 2 × 1 min (Branson Sonicator Model 2210) and centrifuged 15 min at 500 × g (Dynac II, Becton Dickinson). The supernatant was used for enzyme assays.

β-Glucuronidase (EC 3.2.1.31; substrate phenolphthalin mono β-D-glucuronic acid; Sigma, St. Louis, MO), β-glucosidase (EC 3.2.1.21; substrate *p*-nitrophenyl-β-D glucopyranoside; Sigma) and nitroreductase (substrate *m*-nitrobenzoic acid; Sigma) enzyme activities were measured in fecal extracts using the methods detailed by Goldin & Gorbach [45]. The nitroreductase assays were carried out in an anaerobic chamber. Urease (EC 3.5.1.5; substrate urea) activity was measured using the method of Ling et al.[46].

The protein concentration of fecal extracts used in enzymatic assays was determined using the method of Lowry (Sigma), with bovine serum albumin as a standard. All enzyme activities were expressed in units of product produced/mg fecal protein/min.

### Fecal composition analyses

The pH of fecal slurry samples (1 g feces, 10 mL distilled water, mixed with a tissue homogenizer [IKA Labortechnik, Wilmington, NC], 1 min) was measured using a Accumet Model 25 pH meter (Fisher Scientific, Nepean, ON, Canada), and an Orion pH probe (Fisher Scientific). Ammonia concentrations of the fecal enzyme supernatant samples were measured using an Orion Ammonia probe (Fisher Scientific). The short chain fatty acid (SCFA) content of water extracts of fecal material were measured using HPLC techniques [47]. Peaks were identified by comparing retention times to authentic standards purchased from Sigma Chemical Co. (St. Louis, USA).

### Ethics

Informed consent was obtained in writing from all subjects prior to the start of the experiment. The experimental protocol was approved by the McGill University Ethics Committee.

### Statistical analyses

Data were used only when samples from both day 0 and day 56 of a feeding phase were analysed. Analyses of variance (ANOVA) were carried out using the PROC GLM procedure of SAS version 8.02, with milk type (CONT, NAT, SYN), sample day (0 or 56) – main effects, and interactions included in the model. Microbial counts and pH data were analysed as actual counts and hydrogen ion concentration respectively; data in tables and graphs are the log transforms. Inability to produce a sample and contamination (by urine) reduced the number of samples analysed. Legends to tables and figures indicate number of data points contributing to each mean.

Since the initial ANOVA showed no CLA treatment effects, *post facto *the data from the three CLA milk groups (CONT, NAT, SYN) at day 0 and at day 56 were combined, and an ANOVA on the combined data performed (COMBINED DATA).

## Results

Table 1 shows the fatty acid compositions of the milks that were fed. The CONT milk had the lowest levels of *cis*-9, *trans*-11 CLA, and no *trans*-10, *cis*-12 CLA, while the SYN milk had the highest levels of these two fatty acids.

**Table 1:** Milk fat content and fatty acid profile of experimental milks

	Experimental Milk		
	
	CONT	NAT	SYN
Milk fat content, %	3.9	3.9	4.0
Fatty acid, % by weight			
C4:0	4.99	4.44	4.07
C6:0	2.45	1.93	2.18
C8:0	1.40	1.00	1.28
C10:0	2.93	1.87	2.70
C12:0	3.31	2.09	3.02
C14:0	10.79	8.41	10.14
C14:1 *cis*-9	1.07	0.94	0.99
C15:0	1.17	1.00	1.07
C16:0	32.42	20.47	31.18
C16:1 *cis*-9	1.64	1.06	1.50
C17:0	0.58	0.47	0.52
C18:0	9.46	13.00	8.97
C18:1 *trans-*6 + *trans*-7 + *trans*-8	0.30	0.82	0.26
C18:1 *trans*-9	0.23	0.71	0.23
C18:1 *trans*-10	0.34	1.19	0.33
C18:1 *trans*-11	0.92	7.17	0.77
C18:1 *trans*-12	0.49	1.53	0.45
			
C18:1 *cis*-11	0.85	0.65	0.82
C18:1 *cis*-12	0.27	1.39	0.26
C18:1 *cis*-13	0.10	0.10	0.09
C18:1 *trans*-16 + *cis*-14	0.34	.066	0.30
C18:1 *cis*-15	0.09	0.27	0.07
C18:2 *trans*-11, *cis*-15	0.10	0.24	0.13
C18:2 *cis*-9, *cis*-12	1.87	2.23	2.04
C20:0	0.14	0.16	0.16
C18:3 *cis*-9, *cis*-12, *cis*-15	0.38	0.38	0.39
C18:2 *cis*-9, *trans*-11	0.42	2.92	3.28
C18:2 *trans*-10, *cis*-12	n.d.^1^	n.d.	3.03
			
1. n.d. = not detected			

Fifteen subjects successfully finished the entire experiment. Three subjects dropped out mid-way through the interventions. Two subjects, one female and a male, discontinued during the first phase of the clinical trial because of personal reasons. Another female subject discontinued during the second phase of the clinical trial because of pregnancy. Compliance was tested by asking the subjects to return the used and unused milk containers.

The two surveys questioning milk product consumption that were carried out during the course of the experiment (data not presented) indicated that all of the subjects had low daily intakes of milk (skim, 1% fat, 2% fat, whole milk) and dairy products (butter, cheese, yogurt, ice-cream, dairy creamers) as part of their normal diet. During the CLA-milk part of the experiment, subjects were consuming an additional litre of milk per day.

Table 2 is a summary of the microbial analyses of fecal samples collected on days 0 and 56 of the three feeding phases. Within each diet group, there were large inter-subject variations for the enumerated bacteria. Generally, samples contained more total anaerobes than aerobes. ANOVA analyses indicated that the type of milk consumed did not have any effect on the fecal microbial profiles. At the end of the 56 day feeding phases, there were no statistical differences in the number of fecal total aerobes, total anaerobes, enterobacteria and entrococci + streptococci and clostridia compared to the numbers enumerated in samples taken at day 0. However, at the end of 56 days, fecal samples contained significantly fewer lactobacilli and bifidobacteria compared to samples taken at day 0.

**Table 2:** Fecal microbiological profile^1 ^of subjects at Days 0 and 56 of consuming experimental milks

			Statistical Analyses		
			
Bacteria	Day 0	Day 56	Milk Type^2^	Sample Day^3^	MT*SD^4^
Total Aerobes			NS	NS	NS
CONT	8.62	8.56			
NAT	8.50	8.54			
SYN	8.48	8.53			
					
Total Anaerobes			NS	NS	NS
CONT	9.59	10.41			
NAT	11.17	10.72			
SYN	9.50	10.30			
					
Lactobacilli			NS	***	NS
CONT	6.89	5.24			
NAT	6.52	4.99			
SYN	8.07	5.10			
					
Bifidobacteria			NS	***	NS
CONT	7.07	6.44			
NAT	7.06	6.23			
SYN	7.00	6.25			
					
Enterobacteria			NS	NS	NS
CONT	7.52	5.75			
NAT	8.40	5.39			
SYN	7.05	5.37			
					
Entero+Strep			NS	NS	NS
CONT	7.28	4.92			
NAT	6.03	4.76			
SYN	6.43	5.32			
					
Clostridia			NS	NS	NS
CONT	4.84	5.04			
NAT	5.04	5.12			
SYN	4.91	4.97			

1. bacteria counts expressed as log CFU/g wet feces

2. difference due to milk type (CONT vs NAT vs SYN)

3. difference due to sample day (DAY 0 vs DAY 56)

4. interaction – milk type and sample day

**Figure 1:**
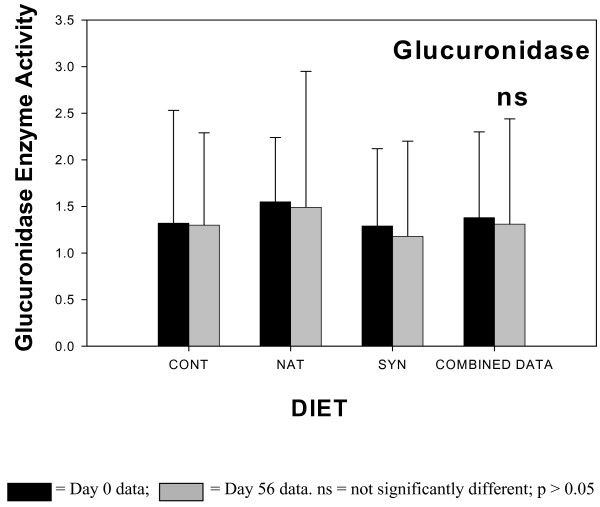
Fecal glucuronidase enzyme activity in subjects consuming milk containing 5 mg/g fat *cis*-9, *trans*-11 CLA – CONT; 32 mg/g fat *cis*-9, *trans*-11 CLA – NAT; 32 mg/g fat *trans*-10, *cis*-12 CLA (32 mg/g fat) and *cis*-9, *trans*-11 CLA – SYN. Number of samples contributing to mean = 12. Data from the 3 different milk groups (CONT, NAT, SYN) for Day 0 and Day 56 = COMBINED DATA. Number of samples contributing to mean = 36. Enzyme activity units – mg phenolphthalein produced/min/mg fecal protein.

**Figure 2:**
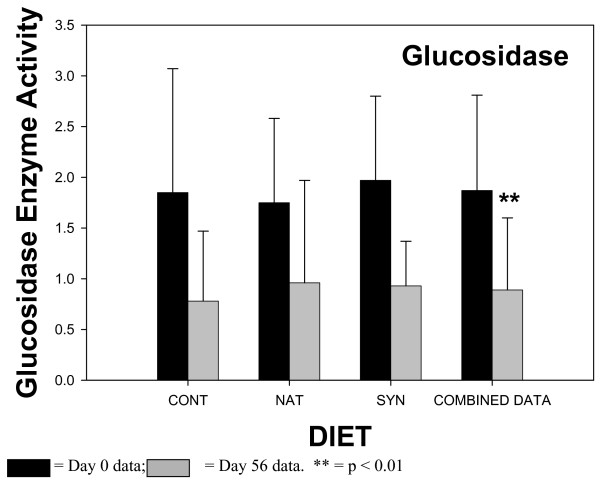
Fecal glucosidase enzyme activity in subjects consuming milk containing 5 mg/g fat *cis*-9, *trans*-11 CLA – CONT; 32 mg/g fat *cis*-9, *trans*-11 CLA – NAT; 32 mg/g fat *trans*-10, *cis*-12 CLA (32 mg/g fat) and *cis*-9, *trans*-11 CLA – SYN. Number of samples contributing to mean = 11. Data from the 3 different milk groups (CONT, NAT, SYN) for Day 0 and Day 56 = COMBINED DATA. Number of samples contributing to mean = 33. Enzyme activity units – mg *p*-nitrophenol produced/min/mg fecal protein.

**Figure 3:**
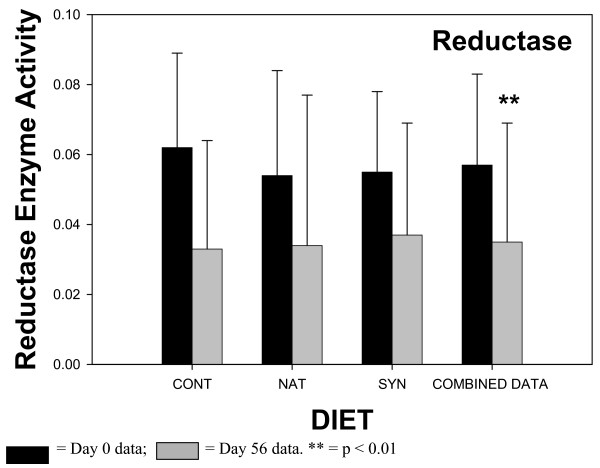
Fecal nitroreductase enzyme activity in subjects consuming milk containing 5 mg/g fat *cis*-9, *trans*-11 CLA – CONT; 32 mg/g fat *cis*-9, *trans*-11 CLA – NAT; 32 mg/g fat *trans*-10, *cis*-12 CLA (32 mg/g fat) and *cis*-9, *trans*-11 CLA – SYN. Number of samples contributing to mean = 8. Data from the 3 different milk groups (CONT, NAT, SYN) for Day 0 and Day 56 = COMBINED DATA. Number of samples contributing to mean = 24. Enzyme activity units – ug *m*-aminobenzoic acidproduced/min/mg fecal protein.

**Figure 4:**
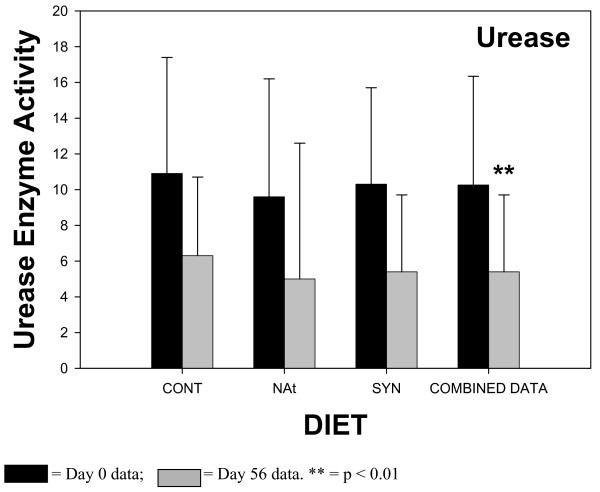
Fecal urease enzyme activity in subjects consuming milk containing 5 mg/g fat *cis*-9, *trans*-11 CLA – CONT; 32 mg/g fat *cis*-9, *trans*-11 CLA – NAT; 32 mg/g fat *trans*-10, cis-12 CLA (32 mg/g fat) and *cis*-9, *trans*-11 CLA – SYN. Number of samples contributing to mean = 12. Data from the 3 different milk groups (CONT, NAT, SYN) for Day 0 and Day 56 = COMBINED DATA. Number of samples contributing to mean = 36. Enzyme activity units – mmoles ammonia produced/min/mg fecal protein.

The results of the fecal enzyme analyses are shown in Figures 1, 2, 3, 4. The enzyme activity for each CLA milk group (CONT, NAT, SYN) is plotted showing values for samples from day 0 and day 56. When ANOVA analyses indicated that there were no statistical differences between any of the different CLA milk groups, all milk group data were combined, and the results of sampling day (0 vs 56) are shown in the fourth pair of bars (COMBINED DATA). This combining of the data had the advantage of increasing the statistical power of the ANOVA test. The activities of β-glucosidase, reductase, and urease in fecal material all significantly declined in samples obtained at day 56 of the feeding phase, compared to samples obtained at day 0 when the COMBINED DATA were analysed. There was no change in fecal β-glucuronidase activity over time.

Fecal pH was not affected by either the kind of CLA milk consumed or the sampling day (0 vs 56). The average pH of all groups was between 7.1 and 7.5. Likewise, ammonia levels measured in fecal samples were not different in subjects consuming different CLA milks or between samples taken at days 0 and 56. Average fecal ammonia concentrations levels ranged from 2–6 mM/mL of fecal extract.

HPLC analyses of fecal samples obtained at day 0 and day 56 of the feeding periods contained acetic acid, propionic acid, butyric acid, and isobutyric acid. The small number of samples analysed prevented rigorous statistical analyses. At day 0, only 50% of the samples analysed contained measurable amounts of lactic acid, but 100% of 56 day samples from the same subjects contained lactic acid.

## Discussion

The make-up of the microbial population that inhabits the human GI tract is influenced by several factors including diet and diet constituents [11,12,22]. Bifidobacteria have been the subject of much research because of the belief that they are probiotic bacteria, and therefore dietary components should be identified that increase bifidobacteria numbers [47-50]. Several studies have shown that significant changes (increases) in fecal bifidobacteria numbers can be achieved by the addition of fructans to the diet [24,51-54].

In our study where subjects were consuming an additional 1 L of milk per day, bifidobacteria numbers significantly declined in all three diet groups over the course of the 56 day feeding phases, perhaps indicating that milk *per se *is affecting this family of bacteria. This is supported by Tanaka [38] who reported bifidobacteria numbers declined (compared to pre-experiment values) in 4 of 10 and 5 of 10 control subjects who were receiving 240 mL/d and 300 mL/d (respectively) of unfermented milk. In addition, we also found that lactobacilli numbers significantly declined, which may have been the result of the consumption of an additional 48 g lactose/day during the experiment.

In this experiment, where adult subjects consumed an additional 1 L of milk during the feeding portions of the experiment, β-glucosidase, nitroreductase and urease activities were all significantly decreased. These effects on fecal enzyme activities occurred in subjects who normally consumed low levels of milk and dairy products. It is not possible at this time to attribute changes in fecal enzyme activity to the changes in the population of one or more fecal bacteria. However, the long term effects of reducing the activities of these enzymes in particular may be desirable.

A reduction in the pH of intestinal digesta has been reported to be desirable [55]. Some feeding trials have reported a lowering of fecal pH [20] but, as we have reported here, other studies have found no changes in fecal pH due to changes in diet [24,26,56]. The use of fecal pH as an indicator of fermentation and acidity in the colon has been questioned [26].

Ammonia in not usually found in high concentrations in fecal material, because any ammonia generated in the intestinal lumen from bacterial breakdown of nitrogenous substances – proteins, urea – is normally quickly absorbed. High levels of intestinal ammonia are not desirable, and it has been shown that the bacterial production of ammonia can be reduced by lowering the pH or adding lactose, glucose or lactulose to the fermentation media [57]. In our subjects, the reduction of the activity of fecal urease did not result in lowered fecal ammonia concentrations.

The effects observed in this feeding trial could be attributed to the increased consumption of milk. At this time, it is not possible say what component of milk caused the observed effects. Our data indicate that consumption of milks with different types and levels of CLAs do not change the intestinal microbiota composition or function. Changes in fecal enzyme activity due to increased milk consumption may have long term health benefits.
